# Development of a Questionnaire for the Reflective Practice of Nursing Involving Invasive Mechanical Ventilation: Assessment of validity and reliability

**DOI:** 10.1002/nop2.212

**Published:** 2018-10-29

**Authors:** Makoto Tsukuda, Atsuko Fukuda, Chiemi Taru, Ikuko Miyawaki

**Affiliations:** ^1^ Department of Nursing, Graduate School of Health Sciences Kobe University Kobe Japan

**Keywords:** factor analysis, Japan, nurses, nursing, psychometrics, questionnaire, reflective practice, reliability, validity

## Abstract

**Aim:**

To develop the Questionnaire for Reflective Practice of Nursing Involving Invasive Mechanical Ventilation (Q‐RPN‐IMV), a Japanese self‐evaluation instrument for ward nurses’ IMV practices.

**Design:**

Cross‐sectional survey.

**Methods:**

Participants were 305 ward nurses from five hospitals in Japan with nursing involving invasive mechanical ventilation. Items concerning the process of nursing practice, including the thought process related to ventilator care, were collected from the literature and observation and interviews with five IMV specialists. Construct validity, concurrent validity, internal consistency and test–retest reliability were tested.

**Results:**

Initially, 141 items were collected and classified into three domains (i.e., observation, assessment and practice). Examination of exploratory factor analysis yielded five factors in the observation domain, six factors in the assessment domain and six factors in the practice domain. The data exhibited internal consistency, stability and concurrent validity. Items of practical content, including thoughts on ventilator care, are useful for preparing educational programmes.

## 1 INTRODUCTION

Worldwide, the number of people who require ventilator‐assisted care has been increasing and this population has unique and variable care needs (Rose et al., 2015). This is especially so in Japan where the rate of ageing of the population is one of the highest in the world (Ministry of Health, Labour, & Welfare, 2016a). Ideally, invasive mechanical ventilation (IMV) care should be administered in an intensive care unit (Guidelines for safe use of ventilator, Japanese Society of Respiratory Care Medicine, 2011). However, advances in medical technology and home healthcare promotion have led to an increasing number of people receiving IMV care in departments other than intensive care units (King, 2015; Lloyd‐Owen et al., 2005). In 2005, there were 5,811 home ventilator‐dependent people on non‐invasive positive pressure ventilation (NPPV) and 611 on IMV. However, in 2015, the number of people on NPPV increased to 2,250 while the number of people on IMV increased to 5,485 (Ministry of Health, Labour, & Welfare, 2016b). Ventilator care may sometimes aim to maintain life in the acute phase or act in synergy with medical treatment for people with incurable diseases, and the required care varies implicitly depending on the situation.

Invasive mechanical ventilation‐associated medical accidents and complications, some of which can be fatal, have become increasingly frequent. Such accidents occur not only due to inadequate ventilator maintenance, but also due to a lack of knowledge and patient observation by medical staff (Aiken, Clarke, Cheung, Sloane, & Silber, 2003; Aiken, Clarke, Sloane, Sochalski, & Silber, 2002; Bond & Raehl, 2007).

In Japan, there are no respiratory therapists with specialist training, unlike the United States; thus, nurses from various backgrounds provide care regarding oxygen therapy, respiratory physiotherapy and artificial respiration therapy to people in hospitals or homes who require therapy (Uzawa, 2006).

Therefore, nurses of all skill levels must be able to administer high‐quality IMV care safely in various settings. Accordingly, nurses often use tools to record ventilator settings chronologically and use checklists to confirm IMV care items, such as checking the cuff pressure.

In addition to these tools, guidelines (on sedation and ventilator weaning bundles, automated weaning systems and preventing ventilator‐associated pneumonia) have supported decision‐making among nurses and resulted in reductions in patient time spent on mechanical ventilation and improved survival rates (Girard & Ely, 2008). Despite these advances, current research highlights significant variation and inconsistency in clinicians’ assessment and practices, which have been shown to have an adverse impact on safety and patient outcomes (Rose & Nelson, 2006). Although some variation is to be expected, it becomes a problem when application of interventions (i.e., weaning protocols) leads to ineffective outcomes for people (Ericsson, Whyte, & Ward, 2007). This suggests that clinical guidelines alone cannot ensure optimum nursing practice for mechanical ventilation.

When nurses provide care during mechanical ventilation, they do not only focus on the criteria provided in guidelines but on patient‐centred information collected from objective physiological and subjective criteria. Especially, less experienced nurses require more encounters with cues (Kydonaki, Huby, Tocher, & Aitken, 2016). Although inexperienced nurses can only record items on existing checklists, they may not understand the implications of those items and may be unable to assess the status of ventilated people. Therefore, these nurses experience uncertainty, stress and anxiety when administering IMV care.

Professional advice and effective and continuous educational programmes are required for nurses who administer IMV care without supervision. In Canada and some other countries, paid personal support workers with minimal appropriate training and education often provide IMV care (Brooks, Gibson, & DeMatteo, 2008).

Although IMV care requires advanced nursing practice skills (Myers, 2013), basic nursing education does not address these skills. Moreover, most nursing educational programmes generally focus only on imparting knowledge on the techniques related to the individual aspects of IMV (Bloos et al., 2009). Consequently, people with various conditions may receive IMV care from nurses who have not received an integrated education that includes all the IMV‐related skills.

Benner, Sutphen, Leonard, Day, and Shulman (2009) explained that “to take action in a given patient‐care situation, the nurse must have a fluent grasp of the relevant medical information and be able to translate it into practical knowledge.” The nursing process, a series of premeditated nursing actions that maintain the best medical environment for people, is a standardized process used to achieve nursing goals. This process comprises four components: assessment, planning, implementation and evaluation. In addition, nurses provide qualitatively and quantitatively adequate nursing care for restoring the health of people whose conditions have changed. Using the nursing process, nurses can plan a course of action to improve a patient's condition (Iyer, Taptich, & Bernocchi‐Losey, 1986).

Given that nursing care is based on the nursing process, the nursing practices associated with IMV should also be analysed accordingly. Therefore, a stepwise and structured education programme based on the nursing process should be provided to nurses, particularly those with little experience in IMV care.

Based on the above literature review, this study aimed to develop an instrument that could clarify the practical nursing process of ventilator care practiced by general nurses in general wards in Japan, where individual nurses need to practice artificial respiratory care according to the situation on each occasion.

In this study, we observed the provision of IMV care by IMV specialists and analysed their assessment processes. Subsequently, we developed a Questionnaire for the Reflective Practice of Nursing Involving Invasive Mechanical Ventilation (Q‐RPN‐IMV) that focuses on the assessment, implementation and evaluation components of the nursing process. The reliability and validity of this questionnaire were also analysed.

This might enable ward nurses to self‐evaluate their own IMV practices and assess the effectiveness of any educational programmes for ward nurses. It could also be used in other countries where nurses working in hospitals or at home will be required to practice IMV care.

## 2 METHODS

This study was conducted to develop the Q‐RPN‐IMV, which covers the practical nursing process of ventilator care in the general ward, including the thought process. The study consisted of item development and validity and reliability testing.

### 2.1 Item development

To develop the items for the Q‐RPN‐IMV, we first reviewed the literature in Japanese and international databases (i.e., PubMed, CINAHL and Japan Medical Abstracts Society database) using the keywords “mechanical ventilation,” “bundle,” “nursing” and “reflective practice.” We also conducted observational and semi‐structured interviews of five IMV specialists, two of whom were certified specialists in critical care nursing and three of whom were certified intensive care nurses. In this way, it covered the process of nursing practice, including the thought process of ventilator care as an item. The literature review and interviews yielded 141 items for the Q‐RPN‐IMV, and these were classified into three domains (i.e., observation, assessment and practice). This list underwent statistical analysis using the survey data.

### 2.2 Validity and reliability testing

#### 2.2.1 Participants

The survey participants were all ward nurses from five acute care general hospitals in Central Japan (N = 305). These hospitals were a convenience sample and had 300–1,000 beds, which showed that they were typical urban, middle‐to‐large‐sized general acute care hospitals. All ward nurses at the five hospitals were contacted about possible participation in this study. The sample size was determined using the general rule for factor analytic procedure that requires a minimum of three respondents per item (Kline, 1998).

#### 2.2.2 Measures

The questionnaire included the following: (a) demographic and professional characteristics; (b) the 141 items (i.e., observation, assessment and practice) for Q‐RPN‐IMV; and (c) the Educational Needs Assessment Tool for Clinical Nurses (Miura & Funashima, 2006).

The demographic and professional characteristics included nursing experience, sex and the number of IMV cases experienced. The 141 items (observation, assessment and practice) for the Q‐RPN‐IMV began with the stem “How often do you practice ventilator nursing care?” with answers recorded on a 5‐point Likert scale ranging from 1 (never) – 5 (always).

The Educational Needs Assessment Tool for Clinical Nurses was used for concurrent validity testing, because this scale is one that yields a higher score for items requiring education regarding professional nursing practice. Regarding items considered necessary for ventilator care, we considered that education was necessary for less frequent practice items; it was the best, currently available instrument among the items of ventilator care. The Educational Needs Assessment Tool for Clinical Nurses consisted of 25 items with five domains measured on a 5‐point Likert scale, with higher scores representing items requiring education regarding professional nursing practice. Cronbach's alpha for this study was 0.94.

### 2.3 Data collection procedure

The researchers explained this study to the nursing managers. When they agreed to participate, the nursing managers were asked to forward questionnaires to the ward nurses. Once the ward nurses agreed to participate in this study, they completed the questionnaires concerning nursing practice, placed their completed questionnaires in a sealed envelope and returned it to the researchers. To evaluate test–retest reliability, ward nurses from five hospitals (N = 152) were asked to complete the questionnaire again after 2 weeks. During the 2‐week interval, participants did not participate in any training, seminars or educational activities.

### 2.4 Data analyses

The Q‐RPN‐IMV was tested for face, factor and concurrent validity. Internal consistency and reproducibility were tested to determine reliability. Each domain was subjected to an exploratory factor analysis using the principal factor method and varimax rotation to test the validity of the models, based on the postulated constructs (i.e., whether all the items for a single factor loaded >0.35) and to confirm that the item loadings were theoretically coherent. Initial factor selection was based on eigenvalues >1.0. After removing items that did not have a loading >0.35 for a given factor, the models were tested via factor analysis, followed by confirmatory factor analysis (Polit & Beck, 2011). The Akaike information criterion (AIC), the comparative fit index (CFI) and the root mean square error of approximation (RMSEA) were used to evaluate the fit of the models to the data (Kääriäinen et al., 2011). With regard to the AIC, the smallest value represents the best classification. RMSEA values <0.05 indicate that a model has a close fit, whereas those between 0.05 – 0.08 indicate a reasonable error when approximating a given structure (Browne & Gudeck, 1993). A CFI >0.9 was assumed to indicate an adequate fit. The final measurement models were selected by examining the four indices of fit and choosing the model with the best indices. Pearson's correlation coefficients for the Q‐RPN‐IMV and the Educational Needs Assessment Tool for Clinical Nurses were calculated to evaluate concurrent validity. We decided that there was a need for education for items with low frequency of practice. Cronbach's *α* coefficient was calculated for each of the subscales to assess internal consistency. Spearman's rank correlation coefficients, which measure the strength of agreement between repeated measurements (Fayers & Machin, 2000), were calculated to evaluate test–retest reliability. All statistical analyses were performed using IBM SPSS 22.0 and Amos 22.0 (IBM Corp., Armonk, NY, USA).

### 2.5 Ethical considerations

Participants were informed of the purpose and methods of the study, risks and benefits of participation, confidentiality of their data and the voluntary nature of participation. Written informed consent was obtained before the interviews for item development and refinement study. The return of the filled anonymous questionnaire was taken as consent to participate in the main study. The study protocol conformed to the tenets of the Declaration of Helsinki (as revised in Edinburgh 2000), and the study process was reviewed and approved by the ethics committee of the Kobe University Graduate School of Health Sciences (approval number: 241).

## 3 RESULTS

### 3.1 Participant characteristics

In total, 432 questionnaires were distributed and 331 returned (response rate: 76.6%). Among them, 305 questionnaires with less than 5% missing data of the total items were used (validity rate: 92.1%). More than half of the participants were nurses who had nursing experience of more than nine years and cared for more than 11 people who had required ventilator care (Table 1).

**Table 1:** Characteristics of the participants (N = 305)

	N (%)
Sex	
Men	21 (6.9)
Women	284 (93.1)
Nursing experience	
<6 years	87 (28.5)
6–10 years	86 (28.2)
>11 years	132 (43.3)
Number of people requiring IMV	
<6	75 (24.5)
6–10	48 (15.7)
>10	182 (59.7)
Number of people requiring IMV per year in the department	
1–3	145 (47.6)
4–10	85 (37.7)
>10	45 (14.8)

Note

IMV: invasive mechanical ventilation.

Data represents N (%).

### 3.2 Item selection and domain development

Among the 141 items collected for the Q‐RPN‐IMV, we classified each item into one of four domains (i.e., observation, assessment, practice and evaluation). Many of the assessment and evaluation items overlapped; therefore, we combined these items into the assessment domain (i.e., observation 26, assessment 67 and practice 48).

#### 3.2.1 Domain 1: IMV care related to observation

The factor analysis of Domain 1 yielded five factors (Table 2). Items 21 and 22 were related to multiple factors. However, we conducted an inspective factor analysis and arranged these items with factor 2. Twenty‐six items that were divided among the five factors had eigenvalues >1.0. Domain 1 consisted of 26 items of five factors.

**Table 2:** Factor analysis of IMV care related to observation (N = 305)

	Factor 1	Factor 2	Factor 3	Factor 4	Factor 5
Factor 1: Essential basic observation					
(7) Breathing sounds	**0.77**	0.09	0.14	−0.03	0.20
(10) Ventilator settings	**0.66**	0.11	0.11	−0.15	−0.03
(14) Contents of the ventilator alarm	**0.64**	0.03	−0.01	0.22	0.07
(11) Ventilator display	**0.57**	0.11	0.14	0.11	0.15
(13) Ventilator alarm setting	**0.52**	0.17	0.13	0.24	0.11
(8) Thoracic movement	**0.50**	0.00	0.01	0.30	0.34
(15) Characteristics of sputum removed per instance of tracheal suction	**0.47**	0.10	0.02	0.35	0.04
(23) Cuff pressure	**0.44**	0.09	0.09	0.16	−0.01
Factor 2: Confirmation of fixed tracheal tube					
(19) Value of endotracheal tube fixing	0.13	**0.83**	0.10	−0.05	0.11
(20) Peeling of tape from the endotracheal tube	0.20	**0.82**	0.07	0.07	−0.01
(18) Redness and ulceration of the lips	0.16	**0.56**	0.10	0.21	0.24
(21) Placement of the tracheostomy tube at midline	0.12	**0.55**	0.13	0.51	−0.05
(2) Sedation level	0.06	**0.54**	0.31	−0.05	0.24
(22) Adequate tracheostomy tube tension	0.14	**0.45**	0.15	0.46	0.01
Factor 3: Confirmation of heating and humidification					
(4) Temperature setting of the heating humidifier	0.16	0.13	**0.91**	0.12	0.19
(5) Water level of the heating humidifier water tank	0.16	0.19	**0.82**	0.00	0.12
(3) Degree of humidification	0.21	0.21	**0.63**	0.14	0.25
Factor 4: Confirmation of positioning and emergency preparation					
(24) Joint range of motion and limb flexibility	0.10	0.07	0.00	**0.53**	0.37
(26) Preparation for re‐intubation	0.12	0.09	0.07	**0.53**	0.40
(16) Positioning	0.36	0.02	0.03	**0.51**	0.22
(25) Placement of BVM at patient's bedside	0.21	0.00	0.10	**0.48**	0.35
Factor 5: Confirmation for predicting complications					
(9) Lung–thorax compliance	0.14	−0.05	0.06	0.26	**0.59**
(6) Radiographic images	0.17	0.09	0.13	0.03	**0.51**
(17) Examination of oral cavity hygiene using a medical penlight	−0.09	0.27	0.13	0.14	**0.48**
(12) Respiratory waveform	0.24	0.09	0.17	0.17	**0.44**
(1) Number of days of mechanical ventilation	−0.06	0.28	0.25	0.08	**0.38**
Sum of squares for factor loading	3.23	2.83	2.27	2.11	2.08
Factor contribution ratio	12.42	10.87	8.74	8.12	8.02
Cumulative factor contribution ratio	12.42	23.29	32.03	40.15	48.16

Note

BVM: bag valve mask.

Bold values indicate factor loading.

#### 3.2.2 Domain 2: IMV care related to assessment

The factor analysis of Domain 2 yielded six factors (Table 3). Item 36 loaded at <0.35 and was removed from the table. Items 12, 49, 50, 51, 53 and 57 were related to multiple factors. To assess these items, we created possible models and conducted an inspective factor analysis. The best‐determined arrangement of the question items was as follows: factor 1 comprised items 51, 53 and 57; factor 3 comprised items 49 and 50; and factor 4 comprised item 12. Sixty‐six items were arranged among the six factors. The six factors (66 items) included had eigenvalues >1.0. Domain 2 comprised 66 items of six factors.

**Table 3:** Factor analysis of IMV care related to assessment (N = 305)

	Factor 1	Factor 2	Factor 3	Factor 4	Factor 5	Factor 6
Factor 1: Assessment of ventilation						
(34) Positive pressure ventilation may cause venous reflex disorder and influence circulatory dynamics	**0.68**	0.23	0.11	0.11	0.17	0.15
(35) Tidal volume can be changed by repositioning	**0.67**	0.16	0.26	0.04	0.20	0.24
(33) Airway pressure and tidal volume can fluctuate when ventilator settings are changed	**0.64**	0.14	0.17	0.03	0.22	0.23
(28) Laterality in thoracic movement indicates differences in right and left pulmonary compliance	**0.58**	0.15	0.19	0.14	0.14	0.17
(30) Reduced thoracic flexibility can inhibit performance of breathing exercises	**0.56**	0.22	0.08	0.25	0.17	0.10
(51) Postural change influences diaphragmatic and thoracic movement	**0.56**	0.11	0.47	0.13	0.20	0.05
(25) Atelectasis and phlegm accumulation occur frequently in the dorsal area of the thorax	**0.52**	0.27	0.14	0.22	0.12	0.02
(55) Reflux of stomach contents might cause ventilator‐associated pneumonia	**0.52**	0.10	0.39	0.17	0.20	0.05
(31) Reduced thoracic flexibility increases difficulty in ventilator withdrawal	**0.49**	0.28	0.09	0.27	0.15	0.07
(48) Tracheal aspiration may cause alveolar collapse	**0.46**	0.15	0.25	0.28	0.22	0.05
(32) Respiratory complications may differ depending on the ventilation style (VCV or PCV)	**0.46**	0.35	−0.01	0.39	0.21	0.01
(26) Change in breathing sounds may indicate alveolar collapse	**0.45**	0.37	0.06	0.33	0.18	0.06
(54) Gravity influences endotracheal secretion	**0.43**	0.14	0.33	0.22	0.17	−0.03
(57) Position changes may change the hang tension in the respiratory circuit	**0.43**	0.11	0.38	0.03	0.13	0.23
(27) Laterality in thoracic movement may indicate one‐lung intubation	**0.42**	0.15	0.11	0.23	0.36	0.17
(1) Long‐term ventilator mounting increases the patient's risk of secondary complications	**0.41**	0.10	0.27	0.15	0.15	0.32
(52) Sitting position decreases the influence of abdominal muscle pressure on the diaphragm	**0.41**	0.16	0.32	0.29	0.24	0.01
(24) Absence of breathing sounds may indicate atelectasis and/or pleural effusion	**0.39**	0.29	0.14	0.09	0.30	0.12
(53) Reduced functional residual volume decreases alveolar gas exchange during breathing exercises in the horizontal dorsal position	**0.38**	0.19	0.23	0.36	0.22	0.09
Factor 2: Assessment of early detection of complications via radiography						
(19) Monitor the degree of atelectasis from transmittance of the lung on the roentgenogram	0.27	**0.82**	0.03	0.06	0.07	0.05
(20) Monitor the degree of pulmonary congestion from transmittance of the lung on the roentgenogram	0.27	**0.82**	−0.02	0.11	0.06	0.04
(22) All ventilation tubes are placed at appropriate positions by checking the tips of various lines on the roentgenogram	0.08	**0.77**	0.05	0.28	0.11	0.02
(21) Monitor the degree of cardiac dilatation by measuring the cardiothoracic ratio on the roentgenogram	0.19	**0.77**	−0.06	0.18	0.08	0.06
(18) Monitor the degree of pleural effusion by checking the blunted costophrenic angle on the roentgenogram	0.25	**0.75**	0.01	0.08	0.01	0.06
(17) Ensure that there is no risk of accidental withdrawal by checking the tip of the endotracheal tube at the carina tracheae on the roentgenogram	0.02	**0.72**	0.05	0.33	0.16	0.07
(16) Ensure that the tip of the endotracheal tube is appropriately placed at the carina tracheae on the roentgenogram to avoid one‐lung intubation	0.02	**0.71**	0.06	0.27	0.13	0.08
(23) Monitor the degree of accidental air ingestion by checking for the presence or absence of gas in the stomach on the roentgenogram	0.10	**0.71**	−0.07	0.42	0.11	0.03
(15) Attempt to detect any changes by comparing the current roentgenogram with the previous photograph	0.18	**0.68**	0.15	0.04	−0.08	0.15
Factor 3: Assessment for predicting complications						
(64) Cuff pressure may damage respiratory tract mucosa, leading to ulcer formation	0.21	−0.02	**0.74**	−0.01	0.15	0.11
(60) Self‐purification capacity of the oral environment decreases among intubated people	0.34	0.00	**0.64**	0.05	0.15	0.17
(67) Ventilator‐associated errors can cause fatal incidents	0.30	−0.04	**0.63**	−0.07	0.04	0.23
(66) Predict possible ventilator‐associated errors	0.29	0.04	**0.58**	0.10	0.10	0.32
(58) Ulcers may form where the endotracheal tube touches the oral mucosa	0.23	0.01	**0.58**	0.05	0.18	0.36
(63) Moisture infiltration into the tape that is used to fix the tube may cause endotracheal tube mobility	−0.04	0.07	**0.57**	0.12	0.21	0.40
(49) Tracheal aspiration may damage the respiratory tract mucosa	0.29	0.02	**0.54**	0.03	0.18	0.13
(62) Attaching fixing tape to loose skin may cause endotracheal tube mobility	−0.05	0.12	**0.54**	0.31	0.18	0.23
(59) Large quantities of bacteria existing in dental plaque are difficult to remove without brushing teeth	0.19	−0.01	**0.47**	0.14	0.18	−0.06
(44) Frequent ventilator alarm may indicate vertical thoracic motion, decreased oxygen saturation or ventilator malfunction	0.36	0.06	**0.46**	0.07	0.28	0.29
(50) Accumulated secretions in the oral cavity, nasal cavity and on the top of the cuff are transported into the lungs via tracheal aspiration while coughing	0.36	0.09	**0.38**	0.26	0.15	−0.13
Factor 4: Assessment for improving the quality of artificial ventilation						
(11) Reduce humidification (while considering phlegm viscosity) when excessive condensation is generated in the corrugated tube	0.24	0.17	0.04	**0.63**	0.10	0.21
(9) Change humidifier settings, especially temperature, when accumulated phlegm is viscous	0.24	0.07	0.08	**0.59**	0.11	0.28
(7) Select a warming humidifier or artificial nose while considering the patient's body temperature	0.05	0.25	−0.07	**0.59**	0.16	0.09
(8) Select a warming humidifier or an artificial nose while considering phlegm quantity and characteristics	0.19	0.19	−0.01	**0.57**	−0.01	0.29
(10) Condensation in the corrugated tube indicates that the air flowing to the patient is sufficiently humidified	0.21	0.10	0.14	**0.55**	0.07	0.18
(61) The endotracheal tube can be bent in the oral cavity even if the tracheal tube remains unchanged in a fixed position of the tracheal tube	−0.03	0.27	0.32	**0.53**	0.18	0.18
(56) If the hip joint axis is beyond the axis of the bed, the diaphragm may be lifted and thoracic movement will be limited	0.12	0.29	0.13	**0.49**	0.13	−0.02
(6) Evaluate the current sedation level according to cough strength during tracheal aspiration	0.16	0.28	0.14	**0.48**	0.24	0.37
(37) The waveform at ventilator exhalation may indicate an air leak in the respiratory circuit	0.09	0.32	0.07	**0.43**	0.21	0.00
(65) Pain during ROM training might influence patient's breathing exercises (e.g., reduced tidal air)	0.19	0.29	0.16	**0.39**	0.13	0.17
(29) Ventilation forced by a respirator causes respiratory muscle fatigue	0.32	0.25	0.10	**0.37**	0.17	−0.07
(12) Evaluate sufficient humidification by monitoring the quantity and characteristics of the phlegm	0.38	0.11	0.22	**0.37**	−0.05	0.43
Factor 5: Assessment for specifying alarm factors						
(39) A high‐pressure circuit alarm may indicate that the patient is fighting the ventilator	0.31	0.11	0.23	0.16	**0.66**	0.24
(45) Fighting the ventilator may cause hypoventilation	0.32	0.16	0.19	0.20	**0.64**	0.27
(46) Fighting the ventilator may increase cardiac load (e.g., exacerbation of oxygenation and severely elevated blood pressure)	0.30	0.17	0.17	0.18	**0.64**	0.34
(38) A high‐pressure circuit alarm may indicate bucking	0.30	0.13	0.30	0.19	**0.63**	0.30
(41) A high‐pressure circuit alarm may indicate a fold in the circuit tube that obstructs the airway	0.31	0.09	0.31	0.19	**0.59**	0.06
(40) A high‐pressure circuit alarm may indicate that the airway is obstructed by secretion	0.31	0.04	0.38	0.12	**0.56**	0.07
(43) A low respiratory rate alarm may indicate apnoea (i.e., over‐sedation)	0.33	0.03	0.38	0.16	**0.56**	0.11
(42) A low‐pressure circuit alarm may indicate a leak in the circuit	0.34	0.04	0.40	0.17	**0.51**	0.07
(47) Fighting the ventilator may be triggered by a problem with the necessity of artificial ventilation for the patient, problems with the patient or respiratory circuit and/or ventilator settings	0.33	0.27	0.14	0.26	**0.48**	0.29
Factor 6: Assessment of safety and comfort of sedation						
(4) Evaluate the level of discomfort based on the sedated patient's respiratory rate and his/her facial expression	0.09	0.04	0.29	0.23	0.27	**0.68**
(3) Evaluate the current sedation level if it reduces the burden on the patient	0.06	0.12	0.17	0.32	0.36	**0.63**
(2) Evaluate the sedation level by calling the patient's name	0.13	0.11	0.23	0.18	0.23	**0.57**
(13) Accumulated water in the corrugated tube may cause accidental swallowing	0.33	0.01	0.32	0.09	0.11	**0.47**
(14) Insufficient water for heating and the flow of overheated air through the tube may cause tracheal burns	0.23	0.04	0.22	0.24	0.09	**0.42**
(5) To avoid delirium, tell sedated people the date and time	0.18	0.31	0.17	0.35	0.16	**0.40**
Sum of squares for factor loadings	7.72	6.88	6.05	5.44	4.92	3.93
Factor contribution ratio	11.69	10.43	9.17	8.24	7.45	5.95
Cumulative factor contribution ratio	11.69	22.12	31.29	39.53	46.98	52.93

Note

PCV: pressure controlled ventilation; ROM: range of motion; VCV: volume control ventilation.

Item 36 (ventilator waveforms indicate synchronization between the patient's breathing rhythm and ventilator) had a low loading and was excluded from the assessment.

Bold values indicate factor loading.

#### 3.2.3 Domain 3: IMV care related to practice

The factor analysis of Domain 3 generated six factors (Table 4). Items 23, 27 and 35 loaded at <0.35 and were removed. Items 30, 34 and 35 were related to multiple factors. To understand these items, we created possible models and conducted an inspective factor analysis. The best‐determined arrangement of question items was as follows: factor 3 comprised items 30, 37 and 38; and factor 4 comprised item 34. A total of 44 items were arranged into six factors and had eigenvalues >1.0. Domain 3 consisted of 44 items of six factors.

**Table 4:** Factor analysis of IMV care related to practice (N = 305)

	Factor 1	Factor 2	Factor 3	Factor 4	Factor 5	Factor 6
Factor 1: Practices for maintaining maximum breathing capacity						
(46) Administer ROM exercises while monitoring tidal volume on the ventilator display to avoid hindering ventilation	**0.82**	0.13	0.07	0.10	0.07	0.08
(45) Ensuring maximal expiratory volume, support the patient's expiration with the palms in accordance with thoracic movement	**0.80**	0.18	0.12	0.09	0.03	0.01
(6) Tap the patient's thorax to locate the diaphragm	**0.75**	0.10	0.09	0.19	0.00	−0.06
(7) Palpate both sides of the thorax to confirm thoracic flexibility	**0.70**	0.27	0.02	0.08	0.12	0.01
(21) Move the shoulder joint in accordance with the patient's breathing while administering ROM exercises to a patient undergoing artificial ventilation	**0.67**	0.15	0.16	0.22	0.04	0.03
(47) If the patient frequently fights the ventilator, change from mechanical ventilation to manual ventilation	**0.67**	0.09	0.12	0.23	0.06	−0.08
(44) Administer ROM exercises daily to prevent contracture	**0.66**	0.12	0.13	−0.10	0.15	0.14
(16) Check for any changes in breathing sounds before and after postural drainage	**0.56**	0.32	0.04	0.12	0.14	0.00
(33) Check cuff pressure before and after position change	**0.52**	0.13	0.05	0.30	−0.11	0.24
(10) Listen to breathing sounds after tracheal aspiration	**0.51**	0.33	0.10	0.09	0.25	0.04
(2) Tell sedated people the date and time	**0.48**	0.18	−0.03	−0.07	0.31	0.25
(25) To prevent respiratory disorders and clear the respiratory tract, roll the patient's body at least 40–60° and change the posture, alternating between left and right	**0.43**	0.34	0.10	0.17	0.16	0.09
(5) Listen to breathing sounds from the dorsal side of the body	**0.41**	0.33	−0.16	0.23	0.25	0.12
Factor 2: Practices for preventing complications						
(18) Adjust the pillow position to avoid extending the patient's neck	0.18	**0.70**	0.05	0.23	0.19	0.03
(17) Change the patient's posture mainly by lifting his/her head	0.15	**0.61**	0.03	0.20	0.18	−0.02
(13) Complete tracheal aspiration (from aspiration catheter insertion to completion) in 15 s	0.13	**0.52**	0.16	−0.11	−0.06	0.18
(15) Perform postural drainage by positioning the lung with pleural effusion or atelectasis at a higher level	0.35	**0.52**	0.13	0.14	0.09	0.07
(14) Record the quantity and characteristics of phlegm after every tracheal aspiration	0.18	**0.50**	0.04	0.03	0.11	0.03
(20) Set aspiration pressure to prevent alveolar collapse	0.30	**0.47**	0.03	0.04	0.00	0.11
(12) Insert the aspiration tube until the tube protrudes 1–2 cm from the tracheal tube tip and stop insertion before the aspiration tube reaches the tracheal bifurcation	0.14	**0.43**	0.17	0.04	0.11	0.17
(8) When ventilator alarms occur, mute the alarm, identify the problem and reset the alarm	0.03	**0.41**	0.19	−0.10	0.29	0.20
(19) Adjust the arm position by placing a pillow under the arms when the patient is in a sitting position	0.30	**0.39**	0.19	0.18	0.17	0.05
(22) Loosen the respiratory circuit before making postural changes	−0.11	**0.39**	0.20	−0.15	0.21	0.29
(26) Brush the teeth of people if possible	0.09	**0.38**	0.03	0.18	0.13	0.24
Factor 3: Practices for safe endotracheal tube fixation						
(41) Two or more staff members should fix the tracheal tubes	0.03	−0.02	**0.66**	−0.15	0.11	−0.02
(39) Fasten fixing tape on the buccal region (maxilla)	0.10	0.32	**0.63**	−0.05	0.09	0.13
(40) After fixing only the tracheal tube with tape, fix the tracheal tube and bite block together with additional tape	0.13	0.15	**0.62**	0.17	0.14	−0.07
(43) When the patient's neck moves because of a change in posture, maintain the root of the tracheal tube by providing manual support	0.19	−0.07	**0.52**	0.21	0.22	0.17
(42) Fix the tracheostomy tube using sufficient strength to allow insertion of one finger on both sides	0.17	0.03	**0.47**	0.23	0.20	0.07
(38) Place skin protection materials on sites that are in contact with the tracheal tubes	0.45	0.19	**0.45**	0.05	−0.04	−0.10
(30) Re‐affix tracheal tubes that are loosened by oral health care	0.04	0.11	**0.42**	−0.04	0.35	0.42
(37) Use remover to remove tracheal tube fixing tapes	0.53	0.11	**0.40**	0.01	−0.06	−0.07
(36) Perform frequent oral suction if the patient demonstrates salivation	0.05	0.26	**0.40**	0.14	−0.02	0.21
Factor 4: Practices for tracheal clearance to prevent VAP						
(29) Before performing oral health care, aspirate in the following order: upper cuff, oral cavity, nasal cavity and trachea	0.34	0.12	0.19	**0.63**	−0.05	0.05
(28) For oral health care, use 200–300 ml of gargle water (tap water) as rinse water	0.36	0.16	0.02	**0.52**	0.08	0.23
(11) Aspirate in the following order: oral cavity, nasal cavity, upper cuff and trachea	0.34	0.23	0.11	**0.40**	0.05	−0.03
(9) Perform oral aspiration before changing the patient's posture	0.31	0.12	−0.11	**0.40**	0.05	0.02
(34) Modify head up‐tilt to ≥35°	0.41	0.38	0.03	**0.37**	−0.03	0.12
Factor 5: Practices for appropriate heating, humidifying and calling the patient's name before and after nursing care						
(4) Refill distilled water in the warming humidifier to prevent the water from emptying	0.01	0.30	0.10	0.02	**0.56**	−0.06
(3) Remove accumulated water in the corrugated tube before postural change	0.17	0.12	0.22	0.07	**0.55**	0.02
(1) Call out to the patient even if he/she is sedated	0.11	0.16	0.21	−0.16	**0.46**	0.31
(24) Maintain a respiratory circuit lower than the tracheal tubes to avoid the transports of water in the circuit into the trachea	0.05	0.26	0.29	0.09	**0.39**	0.15
Factor 6: Practices for appropriate cuff pressure management						
(31) Set adequate cuff pressure using a cuff pressure gauge	−0.09	0.20	0.12	0.06	0.09	**0.57**
(32) Modify the cuff pressure before and after oral health care	0.18	0.29	−0.06	0.27	−0.04	**0.56**
Sum of squares for factor loadings	7.31	4.40	2.99	2.17	1.78	1.73
Factor contribution ratio	15.24	9.18	6.23	4.52	3.71	3.61
Cumulative factor contribution ratio	15.24	24.41	30.65	35.16	38.87	42.48

Note

ROM: range of motion; VAP: ventilator‐associated pneumonia.

The following items had low loading and were excluded from the assessment: (23) Two or more staff members change the patient's body position. (27) Perform oral health care every 6–8 hr. (35) Wash hands before and after touching the respiratory circuit.

Bold values indicate factor loading.

### 3.3 Validity and reliability testing

For each domain, confirmatory factor analysis was performed to investigate the construct validity. Domain 1 (observation) had an AIC = 6,184.51, CFI = 0.80 and RMSEA = 0.08; Domain 2 (assessment) had an AIC = 6,184.51, CFI = 0.74 and RMSEA = 0.075; and Domain 3 (practice) had an AIC = 2,399.71, CFI = 0.76 and RMSEA = 0.068.

Concurrent validity was examined using the Educational Needs Assessment Tool for Clinical Nurses scores (Table 5). Pearson's correlation coefficients were negative for all the items. Accordingly, clinical nurses should be educated on the Q‐RPN‐IMV items, which are infrequently practiced (*r*_s_ = −0.37– −0.46; *p* < 0.001).

**Table 5:** Correlations between the Q‐RPN‐IMV and the Educational Needs Assessment Tool for Clinical Nurses

Educational needs						
Items	Factor 1	Factor 2	Factor 3	Factor 4	Factor 5	Factor 6
	*Domain 1. IMV care related to observation*					
1	−0.191**	−0.257**	−0.289**	−0.343**	−0.319**	
2	−0.234**	−0.306**	−0.288**	−0.325**	−0.276**	
3	−0.183**	−0.267**	−0.277**	−0.307**	−0.289**	
4	−0.103	−0.248**	−0.237**	−0.262**	−0.305**	
5	−0.185**	−0.310**	−0.275**	−0.346**	−0.333**	
6	−0.190**	−0.291**	−0.238**	−0.300**	−0.335**	
7	−0.198**	−0.254**	−0.290**	−0.259**	−0.235**	
8	−0.209**	−0.262**	−0.295**	−0.299**	−0.266**	
9	−0.209**	−0.264**	−0.279**	−0.257**	−0.272**	
10	−0.262**	−0.333**	−0.281**	−0.349**	−0.268**	
	*Domain 2. IMV care related to assessment*					
1	−0.390**	−0.301*	−0.356**	−0.415**	−0.348**	−0.357**
2	−0.368**	−0.201**	−0.336**	−0.377**	−0.369**	−0.383**
3	−0.379**	−0.239**	−0.370**	−0.381**	−0.396**	−0.356**
4	−0.322**	−0.244**	−0.279**	−0.352**	−0.314**	−0.282**
5	−0.373**	−0.294**	−0.318**	−0.404**	−0.324**	−0.346**
6	−0.385**	−0.296**	−0.352**	−0.429**	−0.354**	−0.387**
7	−0.356**	−0.190**	−0.311**	−0.366**	−0.326**	−0.372**
8	−0.365**	−0.215**	−0.330**	−0.369**	−0.333**	−0.364**
9	−0.372**	−0.208**	−0.356**	−0.406**	−0.348**	−0.346**
10	−0.390**	−0.222**	−0.383**	−0.398**	−0.402**	−0.452**
	*Domain 3. IMV care related to practice*					
1	−0.424**	−0.421**	−0.373**	−0.354**	−0.300**	−0.183**
2	−0.348**	−0.420**	−0.292**	−0.298**	−0.300**	−0.270**
3	−0.396**	−0.416**	−0.365**	−0.357**	−0.293**	−0.361**
4	−0.372**	−0.380**	−0.376**	−0.348**	−0.347**	−0.197**
5	−0.434**	−0.421**	−0.318**	−0.328**	−0.316**	−0.298**
6	−0.422**	−0.398**	−0.345**	−0.346**	−0.330**	−0.264**
7	−0.306**	−0.375**	−0.265**	−0.245**	−0.279**	−0.244**
8	−0.335**	−0.396**	−0.219**	−0.287**	−0.284**	−0.283**
9	−0.351**	−0.414**	−0.296**	−0.331**	−0.340**	−0.242**
10	−0.339**	−0.394**	−0.287**	−0.334**	−0.353**	−0.298**

Note

IMV: invasive mechanical ventilation

Educational needs items are as follows: Item 1: Having a clear, fact‐based view of the true nature of problems. Item 2: Prioritizing and solving problems. Item 3: Systematic problem‐solving. Item 4: Assiduous problem‐solving. Item 5: Determining an optimal method for resolving problems based on trial and error. Item 6: Practicing nursing care based on specialized knowledge and expertise. Item 7: Practicing optimal nursing care depending on the patient's needs. Item 8: Relieving people’ distress and anxiety as a top priority. Item 9: Practicing nursing care while considering people’ human rights. Item 10: Practicing nursing care while preventing the risks of possible dangers and expanding professional activities and social skills.

N = 305.

a *p* < 0.05,

b *p* < 0.01: items predicted to show comparative correlation.

For internal consistency, Cronbach's *α* was measured for all factors. Cronbach's *α* coefficients for all factors ranged from 0.52 to 0.93 (Table 6). For reproducibility, the intraclass correlation coefficient for each subscale, which was calculated using data from the 152 participants who returned their test–retest responses, ranged from 0.55 to 0.85 (Table 6).

**Table 6:** Internal consistency (N = 305) and test–retest reliability (N = 152)

	No. of items	Cronbach's alpha	ICC
Observation	26		
Essential basic observation	8	0.81	0.61
Confirmation of fixed tracheal tube	6	0.79	0.73
Confirmation of heating and humidification	3	0.88	0.72
Confirmation of positioning and emergency preparation	4	0.72	0.55
Confirmation for predicting complications	5	0.67	0.76
Assessment	66		
Assessment of ventilation	19	0.93	0.83
Assessment for early detection of complications via radiography	9	0.92	0.85
Assessment for predicting complications	11	0.88	0.72
Assessment for improving the quality of artificial ventilation	12	0.88	0.69
Assessment for specifying alarm factors	9	0.93	0.60
Assessment of safety and comfort of sedation	6	0.84	0.70
Practice	44		
Practices for maintaining maximum breathing capacity	13	0.91	0.83
Practices for preventing complications	11	0.82	0.76
Practices for safe endotracheal tube fixation	9	0.80	0.69
Practices for tracheal clearance to prevent VAP	5	0.75	0.70
Practices for appropriate heating, humidification and calling the patient's name before and after nursing care	4	0.64	0.60
Practices for appropriate cuff pressure management	2	0.52	0.62

Note

ICC: intraclass correlation coefficient; VAP: ventilator‐associated pneumonia

## 4 DISCUSSION

To ensure that IMV care reflects education and supports the practice of high‐quality IMV care, we classified respiratory care items during a nursing intervention process and developed a questionnaire that could confirm the frequency of respiratory care practices performed by clinical nurses. In addition, we classified the enforcement of six practice factors (44 items), six assessment factors (66 items) and five observation factors (26 items).

### 4.1 Reliability of the Q‐RPN‐IMV

With regard to the internal consistency, we calculated Cronbach's *α* coefficients and obtained estimates of 0.64–0.93, indicating a satisfactory consistency and suggesting the potential ability to confirm internal consistency for all the factors except factor 1 (Streiner & Norman, 2003). However, the category “practices for appropriate cuff‐pressure management [and] enforcement of appropriate cuff pressure management” included few items and had a small Cronbach's *α* coefficient of 0.52. However, ventilator‐associated pneumonia‐preventative maintenance, tracheal tube and ventilator volume, which were extremely important factors, were assumed to represent a standard category of practice during respiratory care that included cuff pressure management.

With regard to stable examinations, we calculated the intraclass correlation coefficients between every tested–retested factor to determine the stability of this questionnaire (*r*_s_ = 0.55–0.85; *p* < 0.01). Factors that indicated a firm intraclass correlation coefficient or regular performance in a standard hospital included “assessment of ventilation situation,” “assessment of early detection of complications via radiography” and “practices for maintaining maximum breathing capacity.” The category that frequently received the lowest ranking, “confirmation of positioning and emergency preparation,” was considered to include the items that could be easily addressed.

### 4.2 Validity of the Q‐RPN‐IMV

We observed the respiratory care provided by specialists to ensure that the Q‐RPN‐IMV covered all the items, including ideation. We qualitatively classified these items with regard to the observation, assessment and practice of nursing processes. We confirmed the contents with a respiratory care specialist and subsequently assessed the validity of the instrument. We performed a pretest among the nurses who participated in the study and confirmed the time required to answer an item. The estimated time burden of this necessary measure on each respondent was approximately 30 min. Although the extent of the burden on the respondents was considered, we did not modify the pretest because the responders did not indicate the difficulty of each answer and we deemed all the items indispensable for IMV care.

The questionnaire comprised five observation factors, six assessment factors and six practice factors that had high factor loadings in an exploratory factor analysis. The items were selected via factor analysis confirmation. The observation values were AIC = 2,399.71, CFI = 0.76 and RMSEA = 0.068; the assessment values were AIC = 6,184.51, CFI = 0.74 and RMSEA = 0.075; and the practice values were AIC = 6,184.51, CFI = 0.80 and RMSEA = 0.08. Therefore, this model was not ideal because it followed the assumption of a structure that would link all the factors in each domain. Observation, assessment and practice are implemented consecutively during a nursing intervention process, and therefore, it is necessary to consider the structure that would connect the factors between each domain. However, during the initial stages of this study, we prioritized the determination of the items in each domain and adopted this structure.

With regard to concurrent validity, we identified negative correlations with all the items on the Educational Needs Assessment Tool for Clinical Nurses. The items in this questionnaire covered all the respiratory care practices; the practice frequency and associations related to the items on which education was required were identified, and the criterion‐related validity was determined.

### 4.3 Clinical implementation of the Q‐RPN‐IMV

The possible applications of this questionnaire are unlimited, depending on the method of use. It covers all the practice items necessary for respiratory care and the nursing process, including ideation. Therefore, we determined the meanings and situations appropriately and validated the practice items to ensure that respiratory care practitioners could use this questionnaire during the nursing process. The respiratory care enforcement frequency increased during the retest evaluation because of increased awareness, which likely leads to an increase in the scores of the completed questionnaires. This nurse practicing respiratory care list could be used as a tool for self‐evaluating practice processes. In particular, it could trigger reflection pertaining to the necessity of obtaining new knowledge on the infrequently practiced items.

### 4.4 Educational implementation of the Q‐RPN‐IMV

Checklists and textbooks related to respiratory care education often list only the setting and management of machines and/or items related to care provision (Hong, Chen, & Na, 2012). Using our questionnaire, which addresses all the items related to respiratory care (including ideation), we can confirm the type of care provided by a respiratory care practitioner during a nursing intervention process. Using the questionnaire provided in this study, nurses will be able to safely administer IMV care with confidence, perform self‐evaluations and improve their IMV care skills. The questionnaire can also be used to create an educational programme that supports high‐quality IMV care. Furthermore, educational programme developers, who determine the instructions required for the nursing intervention process, could use this questionnaire for developing respiratory care education depending on the practitioner's needs.

### 4.5 Study limitations

We extracted the items that were identified as representing crucial respiratory care knowledge into the categories of observation, assessment and practice. However, the nursing process comprises a series of steps and is not as simple as a set of divided processes. A nurse's background, which includes experience and departmental area, determines the required skill level. We identified the associations between respiratory factors and the educational programme using a covariance structure analysis. Therefore, it is necessary to examine continuity and factor characteristics.

## 5 CONCLUSIONS

The Q‐RPN‐IMV comprises many items and does not feature an ideal structure. Manuals related to IMV care have been gradually created in bundles. However, IMV care is complicated in the context of nursing practice. Despite good awareness of nurses’ difficulties and the provision of care that is directly linked to people’ lives, a system of educational programmes related to the nursing process has not yet been established. According to our survey, IMV care items could be covered in tandem with nursing practice processes. The findings of this survey represent a major step towards the future establishment of a ventilator‐care education programme for nurses. Respiratory care nurses can use the questionnaire developed in this study for self‐evaluating practice processes. Educational programme developers can control nursing intervention process instructions and use this questionnaire for respiratory care education, depending on the needs of the practitioner. By analysing the structures between factors, education protocols can be created based on continuity and order.

## CONFLICT OF INTEREST

No conflict of interests have been declared by the authors.

## References

[nop2212-bib-0001] Aiken, L. H. , Clarke, S. P. , Cheung, R. B. , Sloane, D. M. , & Silber, J. H. (2003). Educational levels of hospital nurses and surgical patient mortality. Journal of the American Medical Association, 290, 1617–1623. 10.1001/jama.290.12.1617 14506121 PMC3077115

[nop2212-bib-0002] Aiken, L. H. , Clarke, S. P. , Sloane, D. M. , Sochalski, J. , & Silber, J. H. (2002). Hospital nurse staffing and patient mortality, nurse burnout and job dissatisfaction. Journal of the American Medical Association, 288, 1987–1993. 10.1001/jama.288.16.1987 12387650

[nop2212-bib-0003] Benner, P. , Sutphen, M. , Leonard, V. , Day, L. , & Shulman, L. S. (2009). Educating nurses: A call for radical transformation (pp. 53–55). San Francisco, CA: Jossey‐Bass Publication.

[nop2212-bib-0004] Bloos, F. , Müller, S. , Harz, A. , Gugel, M. , Geil, D. , Egerland, K. , & Marx, G. (2009). Effects of staff training on the care of mechanically ventilated people: A prospective cohort study. British Journal of Anaesthesia, 103, 232–237. 10.1093/bja/aep114 19457893

[nop2212-bib-0005] Bond, C. A. , & Raehl, C. L. (2007). Clinical pharmacy services, pharmacy staffing and hospital mortality rates. Pharmacotherapy, 27, 481–493. 10.1592/phco.27.4.481 17381374

[nop2212-bib-0006] Brooks, D. , Gibson, B. , & DeMatteo, D. (2008). Perspectives of personal support workers and ventilator‐users on training needs. Patient Education and Counselling, 71, 244–250. 10.1016/j.pec.2008.01.018 18325719

[nop2212-bib-0007] Browne, M. W. , & Gudeck, R. (1993). Alternative ways of assessing models fit In Bollen K. A., & Long J. S. (Eds.), Testing structure equation models (pp. 136–162). Thousand Oaks, CA: SAGE Publication.

[nop2212-bib-0008] Ericsson, K. A. , Whyte, J. IV , & Ward, P. (2007). Expert performance in nursing: Reviewing research on expertise in nursing within the framework of the expert performance approach. Advances in Nursing Science, 30, E58–E71. 10.1097/00012272-200701000-00014 17299276

[nop2212-bib-0009] Fayers, P. M. , & Machin, D. (2000). Principles of measurements scales In Fayers P. M., & Machin D. (Eds.), Quality of life: The assessments, analysis and interpretation. Oxford, UK: John Wiley & Sons Ltd.

[nop2212-bib-0010] Girard, T. D. , & Ely, W. E. (2008). Protocol driven ventilator weaning: Reviewing the evidence. Clinics in Chest Medicine, 29, 241–252. 10.1016/j.ccm.2008.02.004 18440434

[nop2212-bib-0011] Hong, L. , Chen, L. L. , & Na, L. (2012). Development and evaluation of an appraisal form to assess clinical effectiveness of adult invasive mechanical ventilation systems. Scandinavian Journal of Trauma, Resuscitation and Emergency Medicine, 20, 45 10.1186/1757-7241-20-45 PMC3419130 22747895

[nop2212-bib-0012] Iyer, P. W. , Taptich, B. J. , & Bernocchi‐Losey, D. (1986). Nursing process and nursing diagnosis (pp. 16–17). Philadelphia, PA: W. B. Saunders Company.

[nop2212-bib-0013] Japanese Society of Respiratory Care Medicine (2011). Guidelines for safe use of ventilator (2nd ed.). Retrieved from https://square.umin.ac.jp/jrcm/contents/guide/page06.html (in Japanese)

[nop2212-bib-0014] Kääriäinen, M. , Kanste, O. , Elo, S. , Pölkki, T. , Miettunen, J. , & Kyngäs, H. (2011). Testing and verifying nursing theory by confirmatory factor analysis. Journal of Advanced Nursing, 67, 1163–1172. 10.1111/j.1365-2648.2010.05561.x 21226874

[nop2212-bib-0015] King, A. C. (2015). Long‐term home mechanical ventilation in the United States. Respiratory Care, 57, 921–930. 10.4187/respcare.01741 22663967

[nop2212-bib-0016] Kline, R. B. (1998). Principles and practice of structural equation modeling. New York, NY: Guilford Press.

[nop2212-bib-0017] Kydonaki, K. , Huby, G. , Tocher, J. , & Aitken, L. M. (2016). Understanding nurses’ decision‐making when managing weaning from mechanical ventilation: A study of novice and experienced critical care nurses in Scotland and Greece. Journal of Clinical Nursing, 25, 434–444. 10.1111/jocn.13070 26818369

[nop2212-bib-0018] Lloyd‐Owen, S. J. , Donaldson, G. C. , Ambrosino, N. , Escarabill, J. , Farre, R. , Fauroux, B. , … Wedzicha, J. A. (2005). Patterns of home mechanical ventilation use Europe: Results from the Eurovent survey. European Respiratory Journal, 25, 1025–1031. 10.1183/09031936.05.00066704 15929957

[nop2212-bib-0019] Ministry of Health, Labour and Welfare (2016a). Estimated number of people. Retrieved from http://www8.cao.go.jp/kourei/whitepaper/w-2018/zenbun/pdf/1s1s_01.pdf (in Japanese).

[nop2212-bib-0020] Ministry of Health, Labour and Welfare (2016b). NDB open date. Retrieved from https://www.mhlw.go.jp/file/06-Seisakujouhou-12400000-Hokenkyoku/0000139437.xlsx (in Japanese).

[nop2212-bib-0021] Miura, H. , & Funashima, N. (2006). The development of learning needs assessment tool for clinical nurses. Journal of Research for Nursing Education, 15(1), 7–19. Retrieved from https://www.jstage.jst.go.jp/article/jasne/15/1/15_KJ00004404195/_article/-char/ja (in Japanese).

[nop2212-bib-0022] Myers, T. R. (2013). Thinking outside the box: Moving the respiratory care profession beyond the hospital walls. Respiratory Care, 58, 1377–1385. 10.4187/respcare.02542 23878303

[nop2212-bib-0023] Polit, D. F. , & Beck, C. T. (2011). Nursing research, international edition: Generating and assessing evidence for nursing practice (9th ed.). Philadelphia, PA: Lippincott Williams & Wilkins.

[nop2212-bib-0024] Rose, L. , McKim, D. A. , Katz, S. L. , Leasa, D. , Nonoyama, M. , Pedersen, C. , & CANuVENT Group (2015). Home mechanical ventilation in Canada: A national survey. Respiratory Care, 60, 695–704. 10.4187/respcare.03609 25587173

[nop2212-bib-0025] Rose, L. , & Nelson, S. (2006). Issues in weaning from mechanical ventilation: Literature review. Journal of Advanced Nursing, 54, 73–85. 10.1111/j.1365-2648.2006.03792.x 16553693

[nop2212-bib-0026] Streiner, D. L. , & Norman, G. R. (2003). Health measurement scales: A practical guide to their development and use (3rd ed.). New York, NY: Oxford University Press.

[nop2212-bib-0027] Uzawa, Y. (2006). Current status and issues of respiratory care in Japan. Japanese Journal of Respiratory Care, 23(2), 148–150.

